# Effects of Seasonal Weather on Breeding Phenology and Reproductive Success of Alpine Ptarmigan in Colorado

**DOI:** 10.1371/journal.pone.0158913

**Published:** 2016-07-15

**Authors:** Gregory T. Wann, Cameron L. Aldridge, Clait E. Braun

**Affiliations:** 1 Department of Ecosystem Sciences and Natural Resource Ecology Laboratory, 1231 East Drive, Colorado State University, Fort Collins, CO 80523, United States of America; 2 Department of Ecosystem Sciences and Natural Resource Ecology Laboratory, Colorado State University, in cooperation with U.S. Geological Survey, Fort Collins Science Center, 2150 Centre Avenue, Building C, Fort Collins, CO 80526, United States of America; 3 Grouse Inc., 5572 North Ventana Vista Road, Tucson, AZ 85750, United States of America; Oregon State University, UNITED STATES

## Abstract

Animal populations occurring at high elevations are often assumed to be in peril of extinctions or local extirpations due to elevational-dispersal limitations and thermoregulatory constraints as habitats change and warm. However, long-term monitoring of high-elevation populations is uncommon relative to those occurring at lower elevations, and evidence supporting this assumption is limited. We analyzed 45 years of reproductive data for two Colorado populations of white-tailed ptarmigan (*Lagopus leucura*), an alpine-endemic species with restricted distribution in western North America. Seasonal temperatures measured by the number of growing degree days warmed significantly at our study sites for pre-nesting, nesting, and brood-rearing seasonal periods (mean advance of 8 growing degree days per decade), and both populations advanced their reproductive phenology over the study period based on median hatch dates (median advance of 3.7 and 1.9 days per decade for the northern and southern sites, respectively). Reproductive performance measured by the number of chicks per hen declined significantly at one study site but not the other, and differences between sites may have been due to habitat degradation at one study area. Annual variability in chicks per hen was large at both sites but only weakly related to seasonal weather. An index of precipitation and temperature during the brood-rearing period was the best predictor for reproductive success with warm and dry conditions relating positively to number of chicks per hen. Our results provide evidence for two alpine ptarmigan populations that are remarkably invariant to fluctuations in seasonal weather with respect to reproductive success as measured by number of chicks per hen in the breeding population. These results are surprising given the general perception of alpine animal populations as being highly sensitive to warming temperatures.

## Introduction

Alpine ecosystems are extreme examples of high-elevation habitats and frequently cited as being imperiled due to climate change (e.g., [1, 2]). Animal populations endemic to these systems may be threatened due to constraints to dispersing to higher habitats if physiological limitations to temperature (e.g., [3, 4]) or changing structure and distribution of vegetation (e.g., [5, 6]) make their current habitats unsuitable. Studies in alpine systems have linked climate change to different attributes of animal populations, such as physiology and demography in marmots (*Marmota* spp.) [7], body mass of chamois (*Rupicapra rupicapra*) [8], and distributional shifts in avian populations [9]. However, few studies have demonstrated links between climate change and population declines or extinctions in alpine systems (but see [10] for pika [*Ocotona princeps*] in the Great Basin). The lack of information is undoubtedly due in part to the difficulty of collecting population-level data in alpine systems relative to those occurring at lower elevations. As a result, alpine systems offer opportunities to study populations in extreme yet vulnerable environments.

Animals living in alpine systems must cope with strong seasonal climate changes and short growing seasons when breeding occurs [11, 12]. Species occurring in alpine systems, compared to those in lower elevation habitats with longer growing seasons, are limited in the number of breeding attempts in a breeding season (e.g., [12]), and annual productivity is generally lower in high-elevation systems [13, 14, 15]. Reduced breeding opportunities may cause greater variability in annual fecundity as stochastic events such as extreme spring weather delaying breeding should have larger effects in systems with short versus long breeding seasons [12]. For these reasons, animal populations occurring in alpine systems may be particularly vulnerable to extreme seasonal weather and climate. Warming may also positively impact vertebrate populations in alpine systems if longer growing seasons confer fitness benefits. For example, Ozgul et al. [7] found that yellow-bellied marmots (*Marmota flaviventris*) benefited from increased spring temperatures in Colorado because individuals awakening from hibernation earlier also bred earlier. Earlier breeding was associated with heavier offspring, and heavier offspring survived at higher rates than lighter individuals. Relationships between advanced breeding and fitness may occur in other taxa, such as some avian species, which are known to respond strongly to warming spring temperatures by breeding earlier (e.g., [16]).

The white-tailed ptarmigan (*Lagopus leucura*) is an alpine-endemic species with a restricted distribution in western North America that spends its entire life cycle in the alpine and subalpine [17]. White-tailed ptarmigan (hereafter ptarmigan), when compared to other species in the genus *Lagopus*, have high survivorship and low fecundity [18]. Ptarmigan raise only a single brood in a breeding season but will renest if a nest is lost during egg laying or early incubation [19]. Ptarmigan in Colorado, where our study took place, eat primarily willow (*Salix* spp.) in winter and spring, and various alpine forbs and sedges in summer [20]. The diet of chicks consists almost entirely of invertebrates during the first few weeks of life [21]. Like other species in Tetraoninae, ptarmigan chicks are precocial and incapable of self-thermoregulation during their first weeks of life and must be brooded by hens for warmth (e.g., [22]). Ptarmigan are well adapted for life in the alpine, but there have been recent concerns the species is facing threats from climate warming due to their cold-adapted biology [23].

We analyzed two long-term population data sets for ptarmigan to test the effects of seasonal weather on annual breeding phenology and number of chicks per hen produced in the population (as a measure of reproductive success). Phenology data were obtained in the form of median hatch dates from captured chicks and represents data from successful nests, and reproductive data were based on numbers of chicks observed in the summer per hen in the breeding population. Our aims were to: 1) examine how recent spring warming (temperatures in April through June) has affected breeding phenology, 2) present annual rates of reproductive success based on observed counts of chicks and hens in the breeding population and test for trends in those rates, and 3) test for relationships between annual rates of reproductive success and seasonal weather.

We predicted an advance in timing of breeding in our populations as the relationship between warm springs and timing of nesting is well documented in many avian species (e.g., [24]). Wang et al. [23] also found advancing hatch dates of ptarmigan for a subset of years in one of our populations (1975–1999). Ptarmigan fecundity is known to be adversely affected by spring snow depth [25] and we predicted that precipitation during spring and the pre-laying period would negatively correlate with reproductive success. We also predicted weather would not correlate with reproductive success during the nesting period because hens protect eggs from the abiotic environment during incubation. Precocial willow ptarmigan (*L*. *lagopus*) chicks are also known to be adversely affected by cold and wet conditions during brood rearing [26] which led us to predict that warm and dry conditions would positively correlate with reproductive success during this period. We discuss our results in the context of other alpine and avian studies, as well as implications for future viability of the species.

## Materials and Methods

### Permits

Banding permits were issued by Colorado Parks and Wildlife. Approval for handling wildlife (2009–2012) was provided through Colorado State University's Institutional Animal Care and Use Committee (IACUC).

### Study areas

We studied ptarmigan at two locations in central Colorado beginning in 1968. The Mt. Evans study area (hereafter ME) is roughly 17 km southwest of Idaho Springs (39° 35’ N, 105° 37’ W) and consists of 7.03 km^2^ of alpine habitat with an elevation range between 3535 and 4270 m. Data were collected continuously at ME from 1968 to 2012, with the exception of 1977 and 1999 when field work was limited. The ME study area was open to fall hunting (typically beginning in mid-September and ending in early October) until a permanent closure went into effect in 1994. The Rocky Mountain National Park study area (hereafter RM) consists of 9.11 km^2^ of alpine habitat along Trail Ridge (40° 25’ N, 105° 45’ W) with an elevation range between 3505 and 3688 m. Data were collected from RM continuously from 1968 to 2000, and again in 2011 and 2012. Both study sites have similar habitats characterized as alpine tundra with plant communities consisting of low-growing woody shrubs (e.g., *Salix* spp.), herbaceous forbs (e.g., *Geum rossii*, *Polygonum* spp., *Ranunculus* spp. *Sedum* spp.), sedges (e.g., *Carex* spp.), and grasses (e.g., *Deschampsia* spp., *Poa* spp., *Trisetum* spp.). The lands where research was conducted was managed by the U.S. Forest Service and National Park Service.

### Measuring reproductive success and phenology

Surveys of the study areas occurred in spring (typically the second half of May and first week of June) and summer (typically the second half of August and first week of September). The number of surveys within each season varied by year, although in nearly all years and seasons the study sites were surveyed a minimum of 3 days with 8 or more hours of surveying occurring each day. This amount of time and effort allowed us to survey the extent of area and habitats within the defined study areas. Hens were located and captured in the spring by first locating territorial males using broadcasts of male calls [27]. Males that successfully attracted hens generally stayed in close proximity to their mates, and most hens were found by searching the area in the immediate vicinity of territorial males [27]. Broods were located in the summer by searching suitable habitats (e.g., moist meadows near rock cover) and broadcasting chick distress calls to elicit responses from hens with broods or hens that recently lost broods [27]. Once broods were located we attempted to capture all observed chicks using a noose at the end of a 5-m pole. Captured hens and chicks were marked with numbered State of Colorado aluminum leg bands. Hens also received unique combinations of colored plastic bandettes (between 2 to 4) for individual identification during subsequent resightings. Individual markings of hens greatly reduced the likelihood of double counting broods during surveys. All hens first captured on the study areas in the spring and observed in the summer were considered breeding residents, even if not observed on the study areas in the spring during subsequent years. We considered these hens breeding residents because hens found on the study area in spring exhibit high site fidelity, especially among adults [28]. Dispersal by females to other breeding territories in early spring (both within and outside the study area) is known to occur at low rates at ME and surrounding areas [29]. We assessed potential bias due to sampling effort in our field methodologies which are presented in supporting information (S1 Appendix).

The reproductive measure of interest in this study was the number of chicks produced surviving to the summer count period per hen in the breeding population. Ptarmigan chicks generally remain with females for 8 to 10 weeks [17], although chicks can thermoregulate independently of hens when they reach 3 to 4 weeks of age [22], and may stay with hens beyond 10 weeks if separation does not occur earlier (CEB personal observation). Thus, age at independence is a gradual process and difficult to define [17]. The mean age of broods encountered during summer counts was between 5 to 7 weeks. We used counts of chicks and hens to measure annual variation in reproductive success. The number of chicks per hen was used as an overall measure of reproductive success. The number of chicks per hen was estimated using the total number of chicks observed in summer per hen in the spring population. We used spring hens rather than summer hens because unsuccessful summer hens may disperse long distances after nest or brood failures [29], either into or outside of the study areas. The open nature of ptarmigan populations in summer could introduce bias (e.g., poor reproductive success inside the study areas might lead to higher numbers of unsuccessful hens dispersing outside the study areas and result in a positive bias of reproductive success, and vice versa). It is important to note that the number of chicks available to count in the summer period is the combined product of number of females in the breeding population and, nest, renest, and chick survival probabilities. Hens were not monitored between spring and summer count periods and we could not estimate nest and chick survival. Chicks per hen is a reproductive measure that informs us of how many young per hen in the breeding population survived to the count period after nest and young chick mortality occurred. Previous studies at ME and elsewhere report reproductive rates and survival rates of chicks at various stages [17, 18, 19, 29].

Various body measurements were recorded from captured hens and chicks, including length measurements for primaries 1–10 (measured to the nearest millimeter). Chick ages can be accurately estimated to within a few days based on the length of the most recently replaced primary feather [30], and it was from these measurements that we calculated the date of hatch for each captured chick. An average date of hatch was calculated for every brood by summing the ages of individual chicks within the brood and dividing by the number of chicks assigned to the brood. However, ptarmigan are known to adopt chicks [31], in which case brood averages of date of hatch could be inaccurate if the age of adopted chicks is different from the biological offspring captured with hens. It was possible that hens could be encountered with chicks originating from different broods. If the estimated age of captured chicks differed by 6 days or more from other individuals within the group, we considered those chicks to have originated from a different brood and calculated separate averages. We used date of hatch (reported as the Julian day) as our measure of annual breeding phenology for ptarmigan. The first date of hatch (first brood to hatch in a year) and median date of hatch were response variables in our phenology analysis.

### Covariates

Weather data were obtained from the Niwot Ridge Long Term Ecological Research site. This was the closest location available for our study areas that included the weather variables of interest dating to the beginning of our study. The D1 weather station at Niwot Ridge is at an elevation and topographic position similar to the study locations. ME and RM study areas are 49 km south and 42 km north from the D1 weather station, respectively.

Spring weather data used as explanatory variables for date of hatch included the sum of maximum temperature (warmth sum; WS), cumulative precipitation (CP), and number of growing degree days (GDD). Number of growing degree days was obtained by summing the number of daily growing degrees, which was calculated as: (*T*_*max*_−*T*_*min*_)/2 − *T*_*base*_, where *T*_*max*_ and *T*_*min*_ are the daily maximum and minimum temperatures, and *T*_*base*_ is the base temperature below which plant growth will not occur. We set *T*_*base*_ equal to 0°C and also set a cap equal to 30°C [32]. The choice of temperature explanatory variables was based on previous studies relating weather data to nesting phenology [24, 33, 34]. Past work has shown that weather events occurring up to 2 months prior to the onset of nesting can have strong effects on breeding phenology in avian species [24]. The average nest initiation date in our study populations was estimated to occur on 8 June at ME and 15 June at RM [17]. We used the period 2 months prior to these dates (8 April to 8 June for ME; 15 April to 15 June for RM) to calculate the variables warmth sum, cumulative precipitation, and number of growing degree days.

We examined relationships between reproductive success and weather occurring over seasonal periods for the reproductive analysis. We used weather data for three seasonal periods, including a pre-nesting period covering one month prior to the average onset of egg laying (10 May to 8 June for ME; 17 May to 15 June for RM), a nesting period covering the average times between the onset of egg laying and nest hatching (9 June to 7 July for ME; 16 June to 14 July for RM), and a brood-rearing period covering a two-week period post hatch when chicks are most sensitive to weather (8 July to 21 July for ME; 15 July to 28 July for RM). These periods were defined based on our prior knowledge of reproductive events in the study areas (Braun et al. 1993). Weather variables examined for the seasonal periods included cumulative precipitation (CP), number of growing degree days (GDD), and an index of seasonal wetness and dryness (SIND = GDD/CP). We used a simple naming convention for weather variables presented in models where “S” followed by the season number (1 = spring, 2 = pre-nesting, 3 = nesting, 4 = brood rearing) denotes the period to which we are referring (e.g., S1.GDD is the number of growing degree days during the spring period). A description of the covariates is summarized in Table 1. The spring weather covariates used for the phenology analysis were also considered to affect reproductive success and included in the reproduction analysis.

**Table 1:** Terms used for response variables, covariates, and periods over which weather data were measured. A description for each term is provided. Covariates are presented in the model results with a prefix for the specific period they represent. Study sites were at Mt. Evans (ME) and Trail Ridge at Rocky Mountain National Park (RM) in Colorado.

Term	Type	Description
SITE	Covariate	Categorical variable for study area
CP	Covariate	Cumulative precipitation
GDD	Covariate	Number of growing degree days
SIND	Covariate	Seasonal index (GDD/CP)
P1	Season period	Spring (ME 8 April to 8 June; RM 15 April to 15 June)
P2	Season period	Pre-nesting (ME 10 May to 8 June; RM 17 May to 15 June)
P3	Season period	Nesting (ME 9 June to 7 July; RM 16 June to 14 July)
P4	Season period	Brood rearing (ME 8 July to 21 July; RM 15 July to 28 July)

We were also interested in site, hen age, and density-dependent effects in addition to weather covariates. Site effects were measured using a categorical variable (SITE) to code for each of our two sites. Hen age is an important factor influencing reproductive success in ptarmigan [19], but it could not be directly assessed in our analysis because we modeled annual counts as the response variable. To consider age, we included a covariate that was calculated as the ratio of yearlings to adults in the spring population as a measure of age structure. Density-dependent effects were measured based on the spring density of ptarmigan at our study sites. Intercept only models were used to test the explanatory power of our covariates versus a mean with no annual variation. In the case of weather covariates, we also included site as an additive or interactive effect because weather was examined over slightly different periods (i.e., 7-day difference in first and last dates of seasonal windows) for each site. Log transformations were used on some covariates to better approximate a normal distribution.

### Statistical analysis

Weather variables used in model comparisons (described below) were standardized separately for each site using the transformation $z_{s,y}=(x_{s,y}-{\bar{x}}_{s})/\sigma_{s}$, where the predictor variable *z*_*s*,*y*_ is calculated for every year *y* and site *s*, and the site mean of the untransformed predictor for all years ${\bar{x}}_{s}$ is subtracted from the yearly untransformed predictor *x*_*s*,*y*_ and divided by the site standard deviation *σ*_*s*_. Linear regressions were used to test for temporal trends in weather variables by regressing each variable on year. We used linear regressions to test for temporal trends in breeding phenology by regressing the first and median date of hatch on year for each site. We fit spring covariates to the median date of hatch using regression models separately for each site to test for relationships between phenology and spring weather. Weather variables were left unstandardized for regression models testing temporal and phenological relationships because we wanted to present the coefficients on the scale of the predictors. We define statistical significance at the alpha level of 0.05.

Generalized linear models were used to model the reproductive response variable. The number of chicks per hen was modeled using the number of chicks as the response variable and the log of the number of spring hens as an offset in a negative binomial regression (negative binomial distribution, log link). The presence of the offset in our models indicates we were modeling a rate rather than a count. We used negative binomial regressions because there was evidence of over dispersion in our data, and Vuong’s non-nested hypothesis test [35] indicated that a negative binomial distribution performed better than a Poisson distribution.

An information-theoretic approach was used to select the best models from our candidate model sets using Akaike’s information criterion adjusted for small sample size (AIC_c_ [36]). The number of possible model comparisons was large and, to reduce the number of models tested, we used a hierarchical approach to narrow the number of candidate models. First, we constructed models for all the covariates tested that included the intercept, site, offset, and a single covariate. Covariates that ranked lower than the intercept only model were eliminated from further consideration. The remaining covariates were used to construct the final model comparison set. All combinations of additive and interactive models were constructed with the restriction that collinear covariates (*r* > 0.5) could not occur together in the same model. SITE was also a covariate thought to be important prior to model construction and testing, and all candidate models included this categorical covariate as either an additive or interactive effect. Models in the final candidate set were ranked based on AIC_c_ and the amount of support per model was based on AIC_c_ model weights (*w*_i_).

All statistical analyses were conducted in R [37]. The package VGAM [38] was used to fit negative binomial regression models, and the packages pgirmess [39] and MuMln [40] were used for model ranking and construction of model subsets.

## Results

### Weather variables

The number of growing degree days (GDD) increased over the study span (1968–2012) for every seasonal period tested which included the pre-nesting period (9.5 GDD per decade, *β* = 0.975, SE = 0.358, *P* < 0.01), nesting period (11.9 GDD per decade, *β* = 1.217, SE = 0.599, *P* < 0.05), and brood-rearing period (10.6 GDD per decade, *β* = 1.080, SE = 0.279, *P* < 0.001). Cumulative precipitation and the seasonal index, in contrast, did not change over time for any period. Spring weather used to evaluate breeding phenology had increasing trends for warmth sum (33.7 degrees per decade, *β* = 3.449, SE = 1.501, *P* < 0.05) and number of growing degree days (13.7 GDD per decade, *β* = 1.398, SE = 0.473, *P* < 0.01).

### Breeding phenology

**Fig 1:**
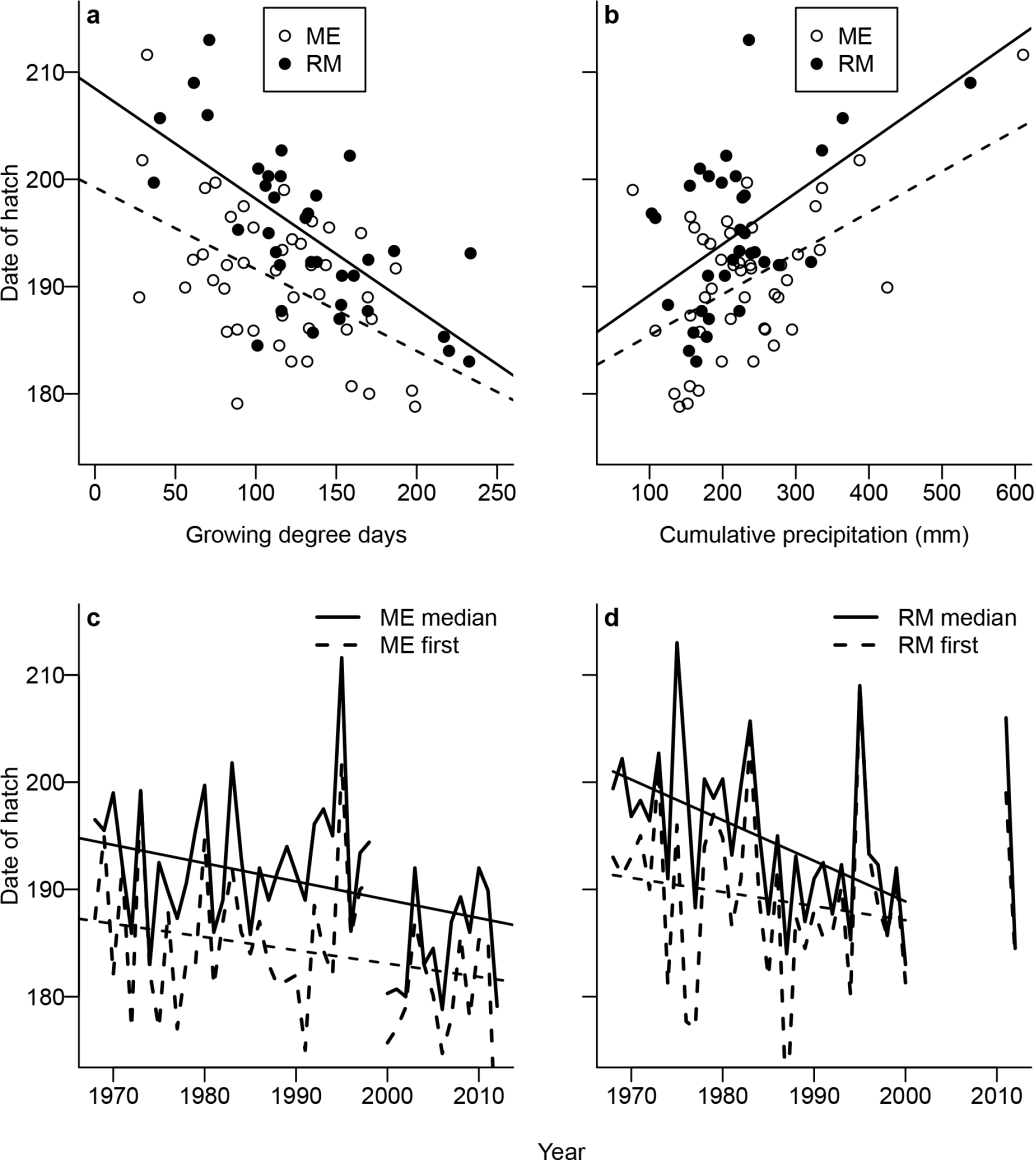
Breeding phenology drivers and temporal trends of white-tailed ptarmigan at Mt. Evans (ME; 1968–2012) and Trail Ridge in Rocky Mountain National Park (RM; 1968–2000, and 2011–2012) in Colorado, USA. Relationships between median date of hatch and number of spring growing degree days (a) and cumulative spring precipitation (b) at ME (open circles, dashed lines) and RM (closed circles, solid lines) are presented along with temporal trends of the first (dashed lines) and median (solid lines) date of hatch at ME (c) and RM (d).

Totals of 583 broods (258 ME, 325 RM) and 1889 chick ages (741 ME, 1148 RM) were used in the phenology analysis. Site-specific and year-specific data are provided in the supporting information (S1 Table). The median date of hatch was negatively related to number of growing degree days at both ME (*β* = -0.076, SE = 0.021, *P* < 0.001; Fig 1A) and RM (*β* = -0.103, SE = 0.019, *P* < 0.001; Fig 1B). The median date of hatch, in contrast, was positively related to spring cumulative precipitation at ME (*β* = 0.038, SE = 0.009, *P* < 0.001) and RM (*β* = 0.048, SE = 0.013, *P* < 0.001; Fig 1B), and median date of hatch was negatively related to warmth sum at both ME (*β* = -0.025, SE = 0.007, *P* < 0.001) and RM (*β* = -0.040, SE = 0.008, *P* < 0.001). The first date of hatch advanced an average of 7.9 days from 1968 to 2012 at ME (1.8 days per decade, *β* = -0.17, SE = 0.08, *P* < 0.05; Fig 1C), but did not advance from 1968 to 2000 at RM (*β* = -0.131, SE = 0.148, *P* = 0.380; Fig 1D). The median date of hatch significantly advanced an average of 8.5 days from 1968 to 2012 at ME (1.9 days per decade, *β* = -0.194, SE = 0.072, *P* < 0.05; Fig 1C), and an average of 12.1 days from 1968 to 2000 at RM (3.7 days per decade, *β* = -0.379, SE = 0.114, *P* < 0.01; Fig 1D).

### Reproductive success

**Fig 2:**
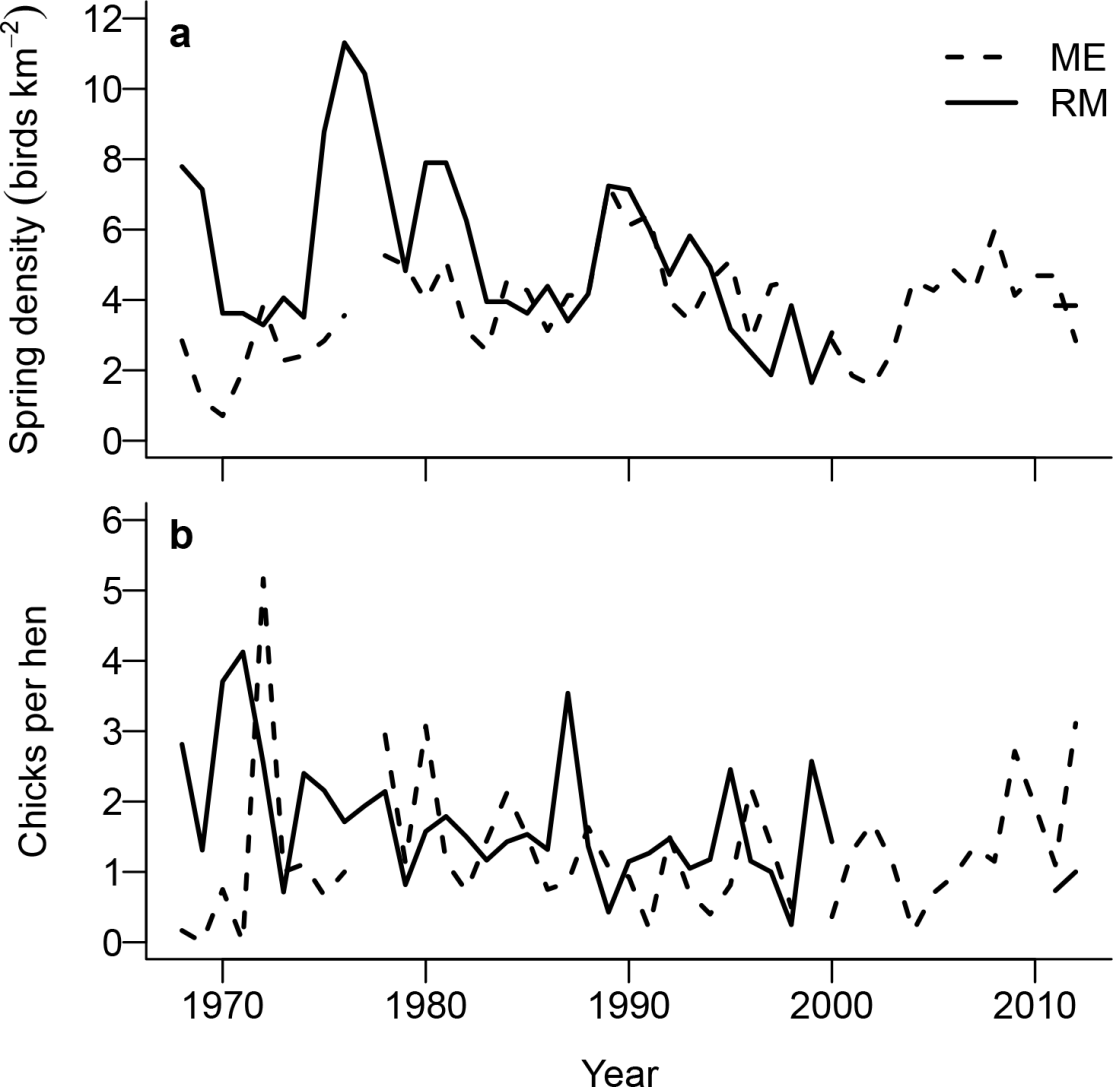
Temporal variation in population density and reproductive success (chicks per hen) of white-tailed ptarmigan in Colorado, USA at Mt. Evans (ME; 1968–2012; black dashed line) and Trail Ridge at Rocky Mountain National Park (RM; 1968–2000, and 2011–2012; black solid line). (a) Spring density of ptarmigan by year. (b) Number of chicks per hen in breeding population by year.

Full spring surveys did not occur at ME in 1977 and 1999, and those years were not used in the reproductive success analysis (S1 Table). The average dates of spring and summer surveys occurred the fourth week of May (ME: $\bar{x}=25$ May, *s* = 10.6 days, range = 1 May to 20 June; RM: $\bar{x}=29$ May, *s* = 12.8 days, range = 1 May to 20 June) and third and fourth week of August (ME: $\bar{x}=19$ August, *s* = 16.2 days, range = 20 July to 20 September; RM: $\bar{x}=27$ August, *s* = 13.5 days, range = 20 July to 20 September). Variation in survey times between years was based primarily on weather in spring and brood ages in summer (i.e., most surveys in summer would occur when broods were old enough to band, generally at 4–6 weeks age). Average age at first capture varied by site (ME: $\bar{x}=49$ days, *s* = 14.5 days, range = 11–91 days; RM: $\bar{x}=41$ days, *s* = 16.9 days, range = 1–88 days). The mean annual number of spring hens observed was 14.7 at ME (SE = 0.883) and 22.8 at RM (SE = 1.932), and the mean number of chicks observed was 18.4 at ME (SE = 2.128) and 38.0 at RM (SE = 4.505). The spring density of ptarmigan declined significantly at RM (*β* = -0.106, SE = 0.040, *P* < 0.05) but marginally increased at ME (*β* = 0.033, SE = 0.016, *P* < 0.05; Fig 2A). Average densities were higher at RM ($\bar{x}=5.18$ birds km^-2^, *s* = 2.32 birds km^-2^, range = 1.65–11.31 birds km^-2^) than ME ($\bar{x}=3.83$ birds km^-2^, *s* = 1.40 birds km^-2^, range = 0.71–7.25 birds km^-2^). The number of chicks per hen declined at RM (*β* = -0.024, SE = 0.008, *P* < 0.01; Fig 2B), but did not change at ME (*β* = 0.012, SE = 0.014, *P* = 0.41; Fig 2B).

**Fig 3:**
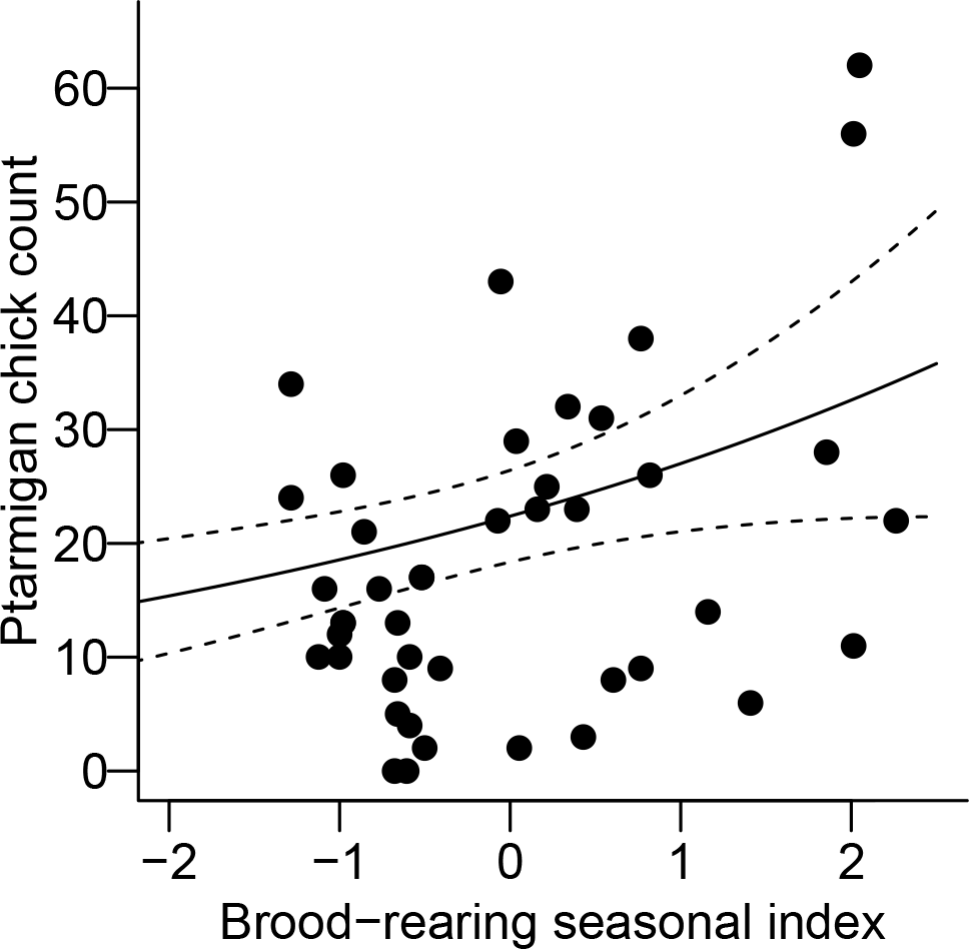
Relationship between brood-period seasonal index and number of white-tailed ptarmigan chicks encountered during summer surveys at Mt. Evans in Colorado, USA. The predictive line was taken from the chicks per hen model receiving the highest AIC_c_ weight (*w*_*i*_) while holding the other parameters in the model at their mean values.

The results from the first stage of model selection eliminated 10 of 15 covariates for number of chicks per hen. The majority of covariates for periods P1-P3 (spring through nesting periods) were generally ranked lower than intercept only models (Table 2 and S2 Table). The model receiving highest support included the seasonal index and number of growing degree days during the brood-rearing period (P4.SIND and P4.GDD) in an additive relationship. The number of chicks per hen was positively related to the seasonal index during the brood-rearing period (P4.SIND; *β* = 0.163, SE = 0.068, *P* < 0.05; Fig 3). Models that included the seasonal index during the brood-rearing period and number of growing degree days during the brood-rearing period were well represented in the top candidate models. The direction of the effects was generally consistent with our *a priori* predictions. Cumulative precipitation during the spring (*β* = -0.075, SE = 0.072, *P* = 0.30) and brood-rearing (*β* = -0.157, SE = 0.071, *P* < 0.05) periods was negatively related to reproductive success, but positively related to reproductive success during the nesting period (*β* = 0.070, SE = 0.071, *P* = 0.30). The relationship between reproductive success and post-hatch growing degree days was negative (*β* = -0.083, SE = 0.071, *P* = 0.24).

**Table 2:** Model selection results for the top ten models for chicks per hen at Mt. Evans and Trail Ridge at Rocky Mountain National Park in Colorado, USA. Number of parameters (*K*), negative log likelihood (LL), difference between the best model and given model (ΔAIC_c_), and AIC_c_ weights (*w*_i_) are provided. Covariates shown include study site (SITE), cumulative precipitation in spring (P1.CP) and brood-rearing period (P4.CP), brood-period seasonal index (P4.SIND), brood-period number of growing degree days (P4.GDD), and brood-period cumulative precipitation (P4.CP).

Model	*K*	LL	Δ AIC_c_	AIC_c_ *w*_i_
SITE + P4.GDD + P4.SIND	6	-305.87	0.00	0.07
SITE + P4.GDD + P4.SIND + P4.SIND x SITE	7	-305.00	0.61	0.05
SITE + P4.SIND	5	-307.44	0.85	0.05
SITE + P4.CP + P4.GDD	6	-306.34	0.93	0.04
SITE + P4.SIND + P4.SIND x SITE	6	-306.43	1.11	0.04
SITE + CSP + P4.GDD + P4.SIND	7	-305.37	1.34	0.04
SITE + CSP + P4.CP + P4.GDD	7	-305.43	1.47	0.03
SITE + P4.CP	5	-307.85	1.67	0.03
SITE + P3.CP + P4.GDD + P4.SIND	7	-305.60	1.80	0.03
SITE + P4.GDD + P4.SIND + P4.GDD x SITE	7	-305.68	1.97	0.03

## Discussion

### Weather and breeding phenology

There was a clear relationship between timing of ptarmigan breeding and spring weather, consistent with other avian studies (e.g., [41]). Breeding occurred earlier during warm springs, but was delayed with increasing precipitation which typically falls in the form of snow in spring in Colorado’s alpine. Ptarmigan populations at ME (1968–2012) and RM (1968–2000) both advanced their timing of breeding roughly 9 and 12 days, respectively, based on the median date of hatch. This corresponds to a breeding advance of 1.9 days per decade at ME and 3.7 days per decade at RM. There were significant warming trends in temperature over this same time period and no change in spring precipitation.

The observed advance in timing of breeding by our populations may have been the result of two possible factors. First, spring warming at our study sites may have caused the observed trend for earlier breeding, given the strong correlation between warm temperatures and timing of breeding. Second, it is possible that changes in nest predation rates could explain the observed changes in phenology if predation of first nests decreased across the study period. Neither hypothesis for the observed trend is mutually exclusive, although we believe that changing spring weather is a much more plausible mechanism for our populations given the close relationship between spring temperature and breeding phenology in ptarmigan, and the observed warming trend in spring precipitation at our study sites. We have no reason to suspect predation rates on first nests changed at either population. Hannon et al. [42] reported timing of nesting of willow ptarmigan was most strongly affected by date of snowmelt, and Wilson and Martin [43] reported that both rock (*Lagopus muta*) and white-tailed ptarmigan responded to warm spring temperatures by advancing their mean breeding dates. Thus, spring temperature is a well-supported driver of timing of breeding in ptarmigan and the plausible cause of earlier nesting observed in our populations.

### Annual variation in reproductive success

Reproductive rates differed between our study sites. We observed no significant trend in chicks per hen across the study period at ME. These results were consistent with annual estimates of recruitment at ME based on mark-recapture data which did not find support for temporal trends [44]. In contrast, reproductive success declined at RM. Reasons for the observed differences between sites are unknown; however, Braun et al. [45] observed heavy browsing of willow along Trail Ridge at RM by elk (*Cervus canadensis*), which they found were correlated with lower ptarmigan breeding densities. Linear temporal declines of willow and forb cover along Trail Ridge have been documented [46]. In addition, willow and forb cover at Trail Ridge is negatively correlated with elk abundance in the park (which steadily increased over the study period; [46]). This offers a possible explanation for the observed decline in reproductive success at the RM study site since breeding densities of ptarmigan have also been declining and brood habitat has likely been degraded. In contrast, breeding densities at ME increased slightly over the study period which was likely due to the hunting closure that went into effect in 1994. Warming winter climate has also been implicated as a driver of population declines at RM. Wang et al. [23] fit population growth models to the observed RM spring count data using winter climate covariates. Results from this study suggested high winter temperatures reduced the population’s growth rate, and the authors did not detect effects from density-dependence. However, the correlative nature of the data did not allow for testing of potential mechanistic drivers relating climate to population growth.

There was little evidence that either median hatch date or spring densities affected reproductive success as measured by chicks per hen as neither covariate ranked better than an intercept only model. Earlier nesting positively affects reproductive success in many species (e.g., [47]), including ptarmigan (e.g., [43]), although it does not necessarily result in higher numbers of fledglings produced (e.g., [42]), or changes in reproductive effort. For example, Wilson and Martin [43] found that white-tailed ptarmigan in the Yukon maintained similar levels of reproductive effort in cold years compared to warm years, with average size of first clutch only declining an average of 1 egg during cold years when breeding was delayed. In contrast, rock ptarmigan at the same study site reduced their reproductive effort of first nests by nearly 3.5 eggs during cold years when breeding was delayed, suggesting different life-history strategies between the two species. These findings indicate reproductive efforts are less variable among years for white-tailed ptarmigan, which also suggests spring weather is unlikely to be strongly correlated with reproductive success. Ludwig et al. [48] predicted that advanced breeding phenology in black grouse (*Tetrao tectrix*) would lead to a trophic mismatch with food at hatch as spring temperatures were increasing at their study sites, but June temperatures (when hatching normally occurred in their study) were not changing. Their hypothesis was empirically supported with long-term data for black grouse. We did not detect asymmetric changes in climate at our sites as both spring and summer temperatures warmed at comparable rates. Thus, the lack of differences in spring and summer warming rates indicates ptarmigan nests and broods should not have been exposed to increasingly cold conditions at our study sites because asymmetric changes in climate did not occur.

The brood-rearing period was the time when climate correlated most strongly with reproductive success in our ptarmigan populations. The seasonal index during the brood-rearing period had a positive relationship with chicks per hen, indicating warm and dry conditions were beneficial during this time. Rock ptarmigan in the Pyrenees had lowest reproductive rates when cumulative precipitation was high during the brood-rearing period [49]. Similarly, Moss et al. [50] reported the number of days with rain after hatching negatively affected the number of chicks raised by capercaillie (*Tetrao urogallus*). The mechanism behind this correlation is likely lost foraging opportunities afforded to chicks in wet weather due to the need to be brooded by hens for warmth [51, 52], because chicks that get wet during cool temperatures could succumb to exposure [53]. It is unclear if higher precipitation during the brood-rearing period can offset the immediate negative impacts if it leads to increased plant productivity, but this may be an important factor to consider in future studies.

### Ptarmigan and future warming

Reproductive success was highly variable across years, but weakly related to weather. Changes in breeding phenology, in contrast, appeared to be strongly linked to seasonal fluctuations in spring weather. However, at our RM site, advancing breeding phenology based on the median date of hatch did not lead to higher reproductive rates, and reproductive success actually declined. It seems ptarmigan respond to local spring weather conditions readily, in terms of timing of breeding activities, but the implication for this plasticity in breeding phenology is not entirely clear. First, if spring weather is a reasonable proxy for timing of food abundance (i.e., plants and insects), ptarmigan populations in undisturbed habitats should be able to cope with variable food abundance given their responsiveness to spring temperatures. In contrast, if the phenology of food is not changing or changing at different rates than spring weather, there is potential for a phenological mismatch to occur between the time ptarmigan raise their young and availability of quality food (e.g., [54]). An experimental study of alpine plants exposed to varying levels of photoperiod and temperature demonstrated that flowering of roughly half of the species tested (23 species) were sensitive to photoperiod, while only a quarter of the species were sensitive to temperature, and a few (5 species) were insensitive to both photoperiod and temperature and flowered as soon as released from snowmelt [55]. This suggests a constraint for many alpine plant species in terms of their flowering phenology with respect to temperature. Thus, ptarmigan breeding may be mistimed if the phenology of forage plants is constrained primarily by photoperiod. Many of the alpine plants consumed by ptarmigan are known [20, 21], but little is known about which of these species is photoperiod versus temperature sensitive with respect to flowering phenology (but see [56] for recent documentation of phenological advances in many alpine plant species in Colorado). Second, it is unknown if earlier nesting observed in our ptarmigan populations is due to simple phenotypic plasticity or if there has been a microevolutionary response to selection. Most studies have been unable to demonstrate an evolutionary response in changes in reproductive phenology and observed changes have generally been attributed to phenotypic plasticity [57].

Other important considerations for predicting ptarmigan population trends in a future with warmer temperatures include habitat effects at both small and large scales. Distributional shifts in areas where species occur are a well-documented response to warming temperatures [58, 59], but factors such as microhabitat are rarely considered. For example, it is commonly assumed that total habitat area decreases monotonically as a function of increasing elevation, which in turn is predicted to compress suitable habitat for mountain species as climate warms [60, 61]. Elsen and Tingley [62] tested this assumption for mountain ranges across the globe and found this pattern was uncommon; many mountain ranges actually had larger surface areas at higher elevations and more complex topographies. This complexity presumably creates larger arrays of microhabitats which are known to be important for some alpine species (e.g. [63]). However, in the Rocky Mountains a monotonically-decreasing pattern was found above mid-elevation areas where ptarmigan typically occur. Nonetheless, this work demonstrates the importance of considering fine-scale habitat availability in addition to larger-scale patterns and careful considerations of the assumption that habitat area decreases with increasing elevation. This is particularly true for species such as white-tailed ptarmigan which occur over a landscape which is already naturally fragmented with reduced areas suitable for occupancy. Recent work by Jackson et al. [64] predicts that summer habitat for ptarmigan populations on Vancouver Island will become severely fragmented in the future with up to 79% declines in patch size of suitable habitat. A similar analysis has not been conducted for Colorado ptarmigan populations, but there are important differences between the areas. Occupied habitats in Colorado are at higher elevations (3350–4268 m [17]) and the alpine areas are more contiguous supporting a larger core population (9,712 km^2^ of suitable habitat and mean densities ranging from 3.5–8.6 birds/km^2^ [65]). In contrast, Vancouver Island is coastal with more isolated patches of alpine at lower elevations (1240–2200 m [17]) and a smaller population (521 km^2^ of suitable habitat [64] and mean densities ranging from 1–2 pairs/km^2^ [Kathy Martin, unpublished estimate]). Thus, ptarmigan in Colorado may have the potential for greater protection from climate warming due to increased opportunities for demographic rescue (e.g., [29]) and other dispersal mechanisms.

In summary, we did not find evidence that ptarmigan reproductive success has been directly negatively impacted by warming at our study sites. The variation in seasonal weather during the breeding period is likely only one of several inter-related factors influencing reproductive success in the ptarmigan populations we studied. There may be important indirect consequences of warming during the breeding season if it facilitates competition with other species for resources used by ptarmigan, or if predation rates are positively affected by warming.

## Supporting Information

S1 Appendix Assessing bias in sampling effort of white-tailed ptarmigan. (DOCX)

S1 Table Reproductive success and phenology data. Data presented in this table include the response variables (first and median date of hatch, number of chicks) and the covariates used to test phenological relationships and build candidate models.
(XLSX)

S2 Table Full candidate model set. (XLSX)

## References

[1] Thompson LG (2000) Ice core evidence for climate change in the tropics: implications for our future. Quat Sci Rev 19: 19–35.

[2] Diaz HF, Grosjean M, Graumlich L (2003) Climate variability and change in high elevation regions: past, present and future. Climatic Change 59: 1–4.

[3] Smith AT (1974) The distribution and dispersal of pikas: influences of behavior and climate. Ecology 55: 1368–1376.

[4] Aublet JF, Festa-Bianchet, Bergero MD, Bassano B (2009) Temperature constraints on foraging behavior of male alpine ibex (*Capra ibex*) in summer. Oecologia 159: 237–247. 10.1007/s00442-008-1198-4 18987895

[5] Dirnböck T, Dullinger S, Grabherr G (2003) A regional impact assessment of climate and land-use change on alpine vegetation. J Biogeogr 30: 401–417.

[6] Wilson SD, Nilsson C (2009) Arctic alpine vegetation change over 20 years. Glob Change Biol 15: 1676–1684.

[7] Ozgul A, Childs DZ, Oli MK, Armitage KB, Blumstein DT, Olson LE, et al (2010) Coupled dynamics of body mass and population growth in response to environmental change. Nature 466: 482–487. 10.1038/nature09210 20651690 PMC5677226

[8] Mason THE, Apollonio M, Chirichella R, Willis SG, Stephens PA (2014) Environmental change and long-term body mass declines in an alpine mammal. Front Zool 11: 69.

[9] Chen IC, Hill JK, Ohlemüller R, Roy DB, Thomas CD (2011) Rapid range shifts of species associated with high levels of climate warming. Science 19: 1024–1026. 10.1126/science.1206432 21852500

[10] Grayson DK (2005) A brief history of Great Basin pikas. J Biogeogr 32: 2103–2111.

[11] Martin K., Holt RF, Thomas DW. (1993) Getting by on high: ecological energetics of arctic and alpine grouse Pages 33–41 in Carey C, Florant GL, Wunder BA, and Horwitz B [eds], Life in the Cold. Westview Press, Boulder, Colorado, USA.

[12] Martin K, Wiebe KL (2004) Coping mechanisms of alpine and arctic breeding birds: extreme weather and limitations to reproductive resilience. Integr Comp Biol 44: 177–185. 10.1093/icb/44.2.177 21680497

[13] Baddyaev AV, Ghalambor CK (2001) Evolution of life histories along elevational gradients: trade-off between parental care and fecundity. Ecology 82: 2948–2960.

[14] Martin M, Camfield AF, Martin K (2009) The demography of an alpine population of savannah sparrows (*Passerculus sandwichensis*). J Field Ornithol 80: 253–264.

[15] Camfield AF, Pearson S, Martin K. (2010) Life history variation between high and low elevation subspecies of horned larks *Eremophila spp*. J Avian Biol. 41: 273–281.

[16] Crick HQP, Sparks TH (1999) Climate change related to egg-laying trends. Nature 399: 423–424.

[17] Martin K, Robb LA, Wilson S, Braun CE (2015) White-tailed ptarmigan (*Lagopus leucura*) In: Poole A (ed) The Birds of North America. Cornell Lab of Ornithology, Ithaca, NY Retrieved from the Birds of North America http://bna.birds.cornell.edu/bna/species/068

[18] Sandercock BK, Martin K, Hannon SJ (2005) Life history strategies in extreme environments: comparative demography of arctic and alpine ptarmigan. Ecology 86: 2176–2186.

[19] Wiebe KL, Martin K (1998) Age-specific patterns of reproduction in white-tailed and willow ptarmigan *Lagopus leucurus* and *L*. *lagopus*. Ibis 140: 14–24.

[20] May TA, Braun CE (1972) Seasonal foods of adult white-tailed ptarmigan in Colorado. J Wildl Manage 36: 1180–1186.

[21] May TA (1970) Seasonal foods of white-tailed ptarmigan in Colorado. Thesis. Colorado State University, Fort Collins, USA.

[22] Pedersen HC, Steen JB (1979) Behavioural thermoregulation in willow ptarmigan chicks *Lagopus lagopus*. Ornis Scandinavica 10: 17–21.

[23] Wang G, Hobbs NT, Giesen KM, Galbraith H, Ojima DS, Braun CE (2002) Relationships between climate and population dynamics of white-tailed ptarmigan Lagopus leucurus in Rocky Mountain National Park, Colorado, USA. Climate Research 23: 81–87.

[24] McCleery RH, Perrins CM (1998) Temperature and egg-laying trends. Nature 391:30–31.

[25] Clarke JA, Johnson RE (1992) The influence of spring snow depth on white-tailed ptarmigan breeding success in the Sierra Nevada. Condor 94: 622–627.

[26] Erikstad KE, Andersen R (1983) The effect of weather on survival, growth rate and feeding time in different sized willow grouse broods. Ornis Scandinavica 14: 249–252.

[27] Braun CE, Schmidt RK, Rogers GE (1973) Census of Colorado white-tailed ptarmigan with tape-recorded calls. J Wildl Manage 37: 90–93.

[28] Giesen KM, Braun CE, May TA (1980) Reproduction and nest-site selection by white-tailed ptarmigan in Colorado. Wilson Bull. 92: 188–199.

[29] Martin K., Stacey PB, Braun CE (2000) Recruitment, dispersal, and demographic rescue in spatially-structured white-tailed ptarmigan populations. Condor 102: 503–516.

[30] Giesen KM, Braun CE (1979) A technique for age determination of juvenile white-tailed ptarmigan. J Wildl Manage 43: 508–511.

[31] Wong MML, Fedy BC, Wilson S, Martin KM (2009) Adoption in rock and white-tailed ptarmigan. Wilson J Ornithol 121: 638–641.

[32] Bowman WD, Seastedt TR. Structure and function of an alpine ecosystem: Niwot Ridge, Colorado New York: Oxford University Press; 2001.

[33] Dunn PO, Winkler DW (1999) Climate change has affected the breeding date of tree swallows throughout North America. Proc Biol Sci 266: 2487–2490. 10693819 10.1098/rspb.1999.0950 PMC1690485

[34] Hussell DJT (2003) Climate change, spring temperatures, and timing of breeding of tree swallows (*Tachycineta bicolor*) in southern Ontario. Auk 3: 607–618.

[35] Vuong QH (1989) Likelihood ratio tests for model selection and non-nested hypotheses. Econometrica 57: 307–333.

[36] Burnham KP, Anderson DR (2002) Model selection and multimodel inference: a practical information-theoretic approach Springer, New York, USA.

[37] R Core Team. (2013) R: a language and environment for statistical computing R Foundation for Statistical Computing, Vienna, Austria http://www.R-project.org/

[38] Yee TW (2010) The VGAM package for categorical data analysis. J Stat Softw 32: 1–34.

[39] Giraudoux P (2013) Pgirmess: data analysis in ecology. R package Version 1.5.8 R Foundation for Statistical Computing, Vienna, Austria http://CRAN.R-project.org/package=pgirmess

[40] Barton K (2014) MuMIn: Multi-model inference R package Version 1.12.1. R Foundation for Statistical Computing, Vienna, Austria http://CRAN.R-project.org/package=MuMIn

[41] Forchhammer MC, Post E, Stenseth NC (1998) Breeding phenology and climate. Nature 391: 29–30.9422504

[42] Hannon SJ, Martin K, Schieck JO (1988) Timing of reproduction in two populations of willow ptarmigan in Northern Canada. Auk 105: 330–338.

[43] Wilson S, Martin K (2010) Variable reproductive effort for two ptarmigan species in response to spring weather in a northern alpine ecosystem. J Avian Biol 41: 319–326.

[44] Wann GT, Aldridge CL, Braun CE (2014) Estimates of annual survival, growth, and recruitment of a white-tailed ptarmigan population in Colorado over 43 years. Popul Ecol 56: 555–567.

[45] Braun CE, Stevens DR, Giesen KM, Melcher CP (1991) Elk, white-tailed ptarmigan and willow relationships: a management dilemma in Rocky Mountain National Park. Transactions of the North American Wildlife and Natural Resources Conference 56: 74–85.

[46] Zeigenfuss LC (2006) Alpine plant community trends on the elk summer range of Rocky Mountain National Park, Colorado: an analysis of existing data U.S. Geological Survey, Fort Collins Science Center, Colorado, USA. Open-File Report 2006–1122.

[47] Perrins CM (1970) The timing of birds’ breeding seasons. Ibis 112: 242–255.

[48] Ludwig GX, Alatalo RV, Helle P, Lindén H, Lindström J, Siitari H (2006) Short- and long-term population dynamical consequences of asymmetric climate change in black grouse. Proc Biol Sci 273: 2009–2016. 16846907 10.1098/rspb.2006.3538 PMC1635476

[49] Novoa C, Besnard A, Brenot JF, Ellison LN (2008) Effect of weather on the reproductive rate of rock ptarmigan *Lagopus muta* in the eastern Pyrenees. Ibis 150: 270–278.

[50] Moss R, Oswald J, Baines D (2008) Climate change and breeding success: decline of the capercaillie in Scotland. J Anim Ecol 70: 47–61.

[51] Marcstrom V (1960) Studies on the physiological and ecological background to the reproduction of the capercaillie (*Tetrao urogallus Lin*.). Wildlife Biol 2: 1–69.

[52] Boag DA (1966) Population attributes of blue grouse in southwestern Alberta. Can J Zool 44: 799–814. 5919293 10.1139/z66-081

[53] Aldridge CL, Boyce MS (2008) Accounting for fitness: combining survival and selection when assessing wildlife-habitat relationships. Isr J Ecol Evol 54: 389–419.

[54] Visser ME, Both C (2005) Shifts in phenology due to global climate change: the need for a yardstick. Proc Biol Sci 282: 2561–2569. 10.1098/rspb.2005.3356 PMC1559974 16321776

[55] Keller F, Körner C (2003) The role of photoperiodism in alpine plant development. Arct Antarct Alp Res 35: 361–368.

[56] CaraDonna PJ, Iler AM, Inouye DW (2014) Shifts in flowering phenology reshape a subalpine plant community. Proc Natl Acad Sci U S A 111: 4916–4921. 10.1073/pnas.1323073111 24639544 PMC3977233

[57] Gienapp P, Teplitsky C, Alho JS, Mills JA, Merillä J (2008) Climate change and evolution: disentangling environmental and genetic responses. Mol Ecol 17: 167–178. 18173499 10.1111/j.1365-294X.2007.03413.x

[58] Parmesan C, Yohe G (2003) A globally coherent fingerprint of climate change impacts across natural systems. Nature 421: 37–42. 12511946 10.1038/nature01286

[59] Chen IC, Hill JK, Ohlemuller R, Roy DB, Thomas CD (2011) Rapid range shifts of species associated with high levels of climate warming. Science 333: 1024–1026. 10.1126/science.1206432 21852500

[60] Tingley MW, Koo MS, Moritz C, Rush AC, Beissinger SR (2012) The push and pull of climate change causes heterogeneous shifts in avian elevational ranges. Glob Change Biol 18: 3279–3290.

[61] La Sorte FA, Jetz W (2010) Projected range contractions of montane biodiversity under global warming. Proc R Soc B 277: 3401–3410. 10.1098/rspb.2010.0612 20534610 PMC2982223

[62] Elsen PR, Tingley MW (2015) Global mountain topography and the fate of montane species under climate change. Nat Clim Chang 5: 772–777.

[63] Wilkening JL, Ray C, Beever EA, Brussard PF (2009) Modeling contemporary range retraction in Great Basin (*Ochotona princeps*) using data on microclimate and microhabitat. Quat Int 235: 77–88.

[64] Jackson MM, Gergel SE, Martin K (2015) Effects of climate change on habitat availability and configuration for an endemic coastal alpine bird. PLoS ONE doi: 10.1371/journal.pone.0142110 PMC4631505 26529306

[65] Hoffman RW (2006) White-tailed ptarmigan (*Lagopus leucura*): a technical conservation assessment. U.S. Department of Agriculture Forest Service, Rocky Mountain Region, Species Conservation Project. 72 pp. Available from: http://www.fs.usda.gov/Internet/FSE_DOCUMENTS/stelprdb5182070.pdf

