# Supplementary material for: Effects of Seasonal Weather on Breeding Phenology and Reproductive Success of Alpine Ptarmigan in Colorado
Source: PLoS One. 2016 Jul 15;11(7):e0158913. doi: 10.1371/journal.pone.0158913 (PMC4946780; doi:10.1371/journal.pone.0158913)
Supplement: S1 Appendix — (DOCX) [file pone.0158913.s001.docx]

**S1 Appendix. Assessing bias in sampling effort of white-tailed ptarmigan.**

**Overview**

We identified one primary way our field methods or methodologies for estimating ptarmigan reproduction could introduce bias to our study. This type of bias involves unequal sampling effort between the spring and summer count periods varying by year. Under this sampling scenario there could be negative or positive bias in our reproductive measures. For example, an intensive search effort in the spring followed by a low search effort in the summer could potentially bias the measure of chicks per hen low due to a decreased probability of finding broods in the summer (i.e. numerator too small). In contrast, a low search effort in the spring followed by an intensive effort in the summer would bias the number of chicks per hen high (i.e. denominator too small). It is important to note that this bias is only a problem if the ratio of sampling effort between sampling periods varies among years, because an unchanging ratio indicates yearly estimates are comparable.

**Methods**

Information on total time spent in the field for each sampling period was not available to assess differences in search effort between spring and summer. As a result, we used a time series of ratios involving the number of individually identifiable hens and total number of observations per hen between count periods to assess if sampling effort varied temporally in our study. To do this we first calculated a spring ratio ($SPR$) as the ratio of number of individual hens observed in the spring ($NSpHens$) to the total number of observations of all individually identifiable hens observed in the spring ($OSpHens$) for a given year *t*:

${SPR}_{t}= {OSpHens}_{t}/{NSpHens}_{t}$ (1)

Note that a ratio of $SPR = 1$ indicates the lowest possible sampling effort. This was also done to calculate a summer ratio ($SUR$):

${SUR}_{t}= {OSuHens}_{t}/{NSuHens}_{t}$ (2)

Since our study evaluated annual reproductive rates, we were interested if the ratio between $SPR$ and $SUR$ varied by year. To assess this, we calculated annual sampling effort ($ASE$) as the ratio of $SPR$ to $SUR$ for a given year *t*:

${ASE}_{t}= {SPR}_{t}/{SUR}_{t}$ (3)

Note that values of $ASE$ > 1 indicate a greater spring sampling effort while $ASE$ < 1 indicates a greater summer sampling effort. To evaluate if an annual trend occurred in sampling effort we used a simple linear regression and regressed $ASE$ on year. A coefficient estimate of year that is close to 0 (*P* > 0.1) would indicate a lack of trend in$ASE$.

**Results**

There was no evidence for a temporal change in $ASE$ at either ME (*β*_year_ = 0.000, SE = 0.003, *P* = 0.910) or RM (*β*_year_ = 0.003, SE = 0.003, *P* = 0.392). $ASE$ was randomly distributed around 1 indicating no apparent systematic sampling bias between seasons.
